# Parent’s sociodemographic factors, physical activity and active commuting are predictors of independent mobility to school

**DOI:** 10.1186/s12942-021-00280-2

**Published:** 2021-06-06

**Authors:** F. Rodríguez-Rodríguez, P. Gálvez-Fernández, F. J. Huertas-Delgado, M. J. Aranda-Balboa, R. G. Saucedo-Araujo, M. Herrador-Colmenero

**Affiliations:** 1 IRyS Group, School of Physical Education, Pontificia Universidad Católica de Valparaíso, Valparaíso, Chile; 2 Department of Physical Education and Sports, PROFITH “PROmoting FITness and Health Through Physical Activity” Research Group, Sport and Health University Research Institute (iMUDS), Faculty of Sport Sciences, University of Granada, Granada, Spain; 3 Teacher Training Centre La Inmaculada. University of Granada, Granada, Spain

**Keywords:** Active transport, Active behaviour, Schoolchildren, Youth, Autonomy, Family

## Abstract

**Background:**

Independent mobility (IM) provides young people with many opportunities to increase their autonomy and physical activity (PA). This study aimed to analyse whether the parent’s PA, active commuting to work and sociodemographic factors serve as predictors of IM to school in children and adolescents.

**Methods:**

A total of 684 parents (52.8% mothers) and their offspring (56.4% girls) were included in this study, which was performed in Granada (Spain) and Valparaíso (Chile). The parents self-reported their sociodemographic characteristics, PA and mode of commuting to work. The mode of commuting to and from school and the offspring accompaniment mode were reported. T-test and chi-square test were used to study quantitative and qualitative differences by parental gender, respectively. Binary logistic regression models (odds ratio = OR) and stepwise analysis were performed to study the association between the parents’ sociodemographic variables and IM to school.

**Results:**

Adolescents showed higher IM to school than children (58.9% vs 40.2%; p < 0.001). No car availability and shorter distance to work were positively associated with higher IM to school in children (OR = 2.22 and 2.29, respectively). Mothers' lower salary/month (OR = 2.75), no car availability (OR = 3.17), and mother passive commuting to work (OR = 2.61) were positively associated with higher IM to school in adolescents. The main predictor of IM to school in children and adolescents was no car availability (OR = 6.53).

**Conclusion:**

Parental sociodemographic factors, such as salary, distance to work and car availability, were associated more strongly with IM than parental PA and active commuting to work.

**Supplementary Information:**

The online version contains supplementary material available at 10.1186/s12942-021-00280-2.

## Background

Regular physical activity (PA) has been associated with physical and psychosocial health and well-being benefits in children and adolescents [1]. These benefits are obtained by adopting the PA recommendations of 60 min per day in moderate-to-vigorous PA (MVPA) in children and adolescents and 150 min per week in MVPA or 75 min at vigorous PA in adults [2, 3]. Currently, most children (54–80%), adolescents (80.3%), and adults (31.1%) worldwide (included Chilean and Spanish) do not meet the current recommendations [4–6]. Additionally, the evidence has indicated that the practice of PA in childhood is transferred to adulthood [7]. Socio-ecological models are commonly used to explain the determinants of healthy behaviours. Sallis et al. [8] proposed an ecological model of active living comprising four domains (recreation, active commuting, occupation, and household) with the aim of increasing PA. Consequently, active commuting to school (ACS), walking or cycling, is related to an active living style in children and adolescents and has been presented as a good opportunity to increase daily PA levels [9]. Thus, ACS becomes a priority to promote this active behaviour in children and adolescents. This especially since the support of families, particularly parents, has been identified as an important factor to provide children with positive examples of healthy and active behaviours [10]. In this regard, the rates of ACS ranged between 20 and 60% in Chilean and Spanish youth respectively [4].

ACS also offers opportunities for independent mobility (IM) which is defined as the freedom that children to travel around their home neighbourhood or city without adult supervision [11]. IM provides children and adolescents many opportunities to increase PA levels [12–14]. Being allowed to travel unsupervised generates greater opportunities for children to be active [11]. Additionally, IM provides psychosocial, cognitive, and personal developmental benefits in the form of social interactions with peers, spatial and traffic safety skills to navigate public spaces, and decision-making maturity [15–17]. Children engaging in IM perceived their home neighbourhoods to be safer than those who did not engage in IM [18]. Individual factors such as a child’s age, gender and confidence in their abilities [19] are crucial in the negotiation of children’s freedom to move and commuting independently. The age at which children are granted IM has increased compared with younger age groups. In addition, several studies have consistently demonstrated that IM increases as age increases [20–22]. According to this, IM could be largely dependent on the parents especially in young children, where parental permission can intervene in the increase or decrease of IM [19, 23, 24]. Nevertheless, other less modifiable family factors could affect IM, such as the parents’ age, gender, PA level, or socioeconomic status [18], but the evidence is not clear yet in identifying the factors that most affect IM in children and adolescents. Currently, around 47–60% of Spanish children and adolescents commute independently [18], and in Chilean children no information exists yet. According to our knowledge, to date, very few studies have analysed the gender-specific association of IM between parents and offspring [23], either the association between socioeconomic level and IM [25]. However, more studies found evidence that if household car ownership increased, the likelihood of IM decreased [21, 26–28]. In addition, children’s school hours usually coincide with their parents’ working hours, causing the parents to escort their children to school for convenience, reducing IM to school [29]. Furthermore, a positive association was found between increased working hours and longer distances to work of mothers and IM to school because they are less likely to chauffeur their children [30]. Additionally, parents’ concerns, such as traffic safety and crime-related safety, affect IM to school negatively [31].

In summary, parental factors have been studied independently but not considering both factors at the same time [18, 19, 23, 25]. This is important, since behaviour is regulated by several factors that combine have more often been analysed separately than considering their complex interrelations [32] that happen in real life. These less modifiable parents or family factors that affect IM to school must be analysed to develop more effective strategies and interventions from social, educational, and public health perspectives that would benefit children and adolescents. Therefore, this study aimed to analyse which of the parental factors, sociodemographic, PA or active commuting to work can explain better the IM in children and adolescents.

## Materials and methods

### Study design and participants

**Fig. 1:**
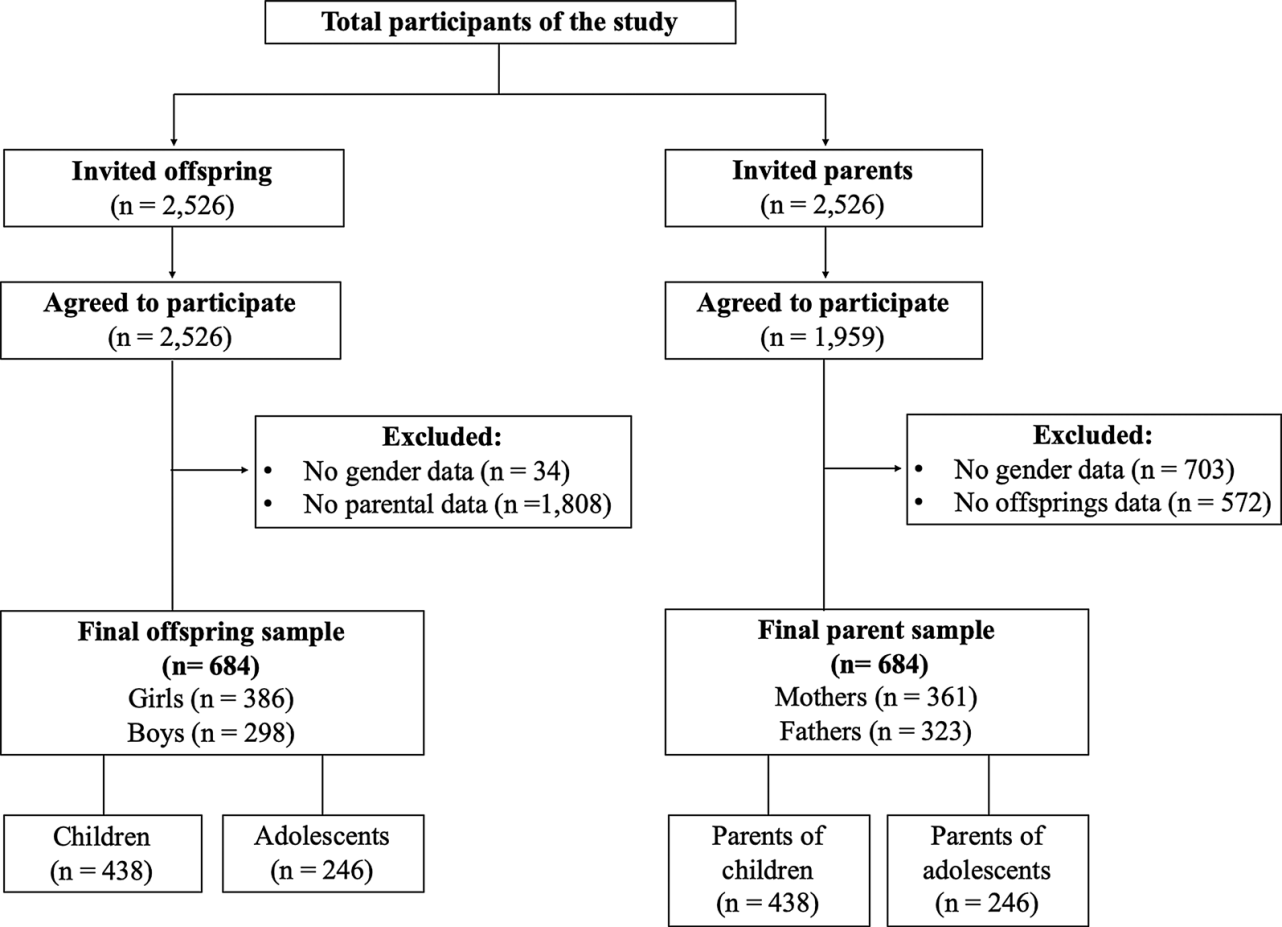
Flow chart of the participants

The data were obtained from the “Cycle and Walk to School” (PACO, for its Spanish acronym), a cross-sectional and quasi-experimental study focused on promoting PA levels and ACS. The study was performed in Granada (Spain) and Valparaíso (Chile) between 2015 and 2018, regularly in spring. Twenty schools were invited to participate in the study as a non-randomized sample. The participants attended 15 schools in Granada (n = 494) and five schools in Valparaíso (n = 192). The sampling has been obtained for convenience, where initially 5052 children and adolescents and their parents were invited to participate, with a response rate of 88.7% (n = 4485). After pairing the parents and offspring and excluding the students who did not report their gender, 684 data dyads of parents (52.8% mothers) and their respective offspring (56.4% girls) were considered (Fig. 1). The ages (mean ± standard deviation) of the participants in each group were as follows: parents, 43.4 ± 6.5 years; children, 9.7 ± 1.7 years; adolescents, 14.0 ± 1.7 years (Tables 1 and 2).

**Table 1:** Sociodemographic factors, mode of commuting, and PA between mothers and fathers

	Overall (n = 684)		Mothers (n = 361)		Fathers (n = 323)		*p-value*
	N	(%)	N	(%)	N	(%)	
Sociodemographic factors							
Age (Mean ± SD)	43.4	± 6.5	42.7	± 6.5	45.7	± 6.0	0.094
Educational level							
Low-medium education	300	(45.9)	125	(35.1)	175	(58.7)	< 0.001
Higher education	354	(54.1)	231	(64.9)	123	(41.3)	
Salary/month							
< 1000 €	212	(55.9)	159	(60.5)	53	(45.7)	0.004
≥ 1000 €	167	(44.1)	104	(39.5)	63	(54.3)	
Car availability							
None	123	(21.4)	36	(11.5)	87	(33.3)	< 0.001
One or more	451	(78.6)	278	(88.5)	173	(66.5)	
Family affluence^a^							
Medium	18	(4.7)	14	(4.8)	4	(4.3)	0.608
High	363	(95.3)	275	(95.2)	88	(95.7)	
Distance of commuting to work							
< 1 km	73	(16.6)	46	(19.1)	27	(11.9)	0.021
≥ 1 km	395	(84.4)	195	(80.9)	200	(88.1)	
Recommendation for MVPA							
< 150 min in MVPA	195	(37.6)	93	(29.9)	102	(49.0)	< 0.001
≥ 150 min in MVPA	324	(62.4)	218	(70.1)	106	(51.0)	< 0.001
Mode of commuting							
Active commuting to work	250	(36.7)	137	(38.0)	113	(35.2)	0.254
Passive commuting to work	432	(63.3)	224	(62.0)	208	(64.8)	

*MVPA* moderate-vigorous physical activity, *SD* standard deviation

^a^No low level in Family affluence scale was found

**Table 2:** Sociodemographic factors, mode of commuting, distance and accompaniment to school between children and adolescents

	Overall (n = 684)		Children (n = 438)		Adolescents (n = 246)		*p-value*
	N	(%)	N	(%)	N	(%)	
Sociodemographic factors							
Age (Mean ± SD)	11.3 ± 2.7		9.7 ± 1.7		14.0 ± 1.7		< 0.001^a^
Gender							
Girls	386	(56.4)	243	(55.5)	143	(58.1)	0.521
Boys	298	(43.6)	195	(44.5)	103	(41.9)	
Mode of commuting (n = 673)							
Active	263	(39.1)	169	(39.0)	94	(39.2)	0.518
Passive	410	(60.9)	264	(61.0)	146	(60.8)	
Distance to school (n = 684)							
< 1 km	477	(69.7)	275	(62.8)	202	(82.1)	< 0.001^a^
≥ 1 km	207	(30.3)	163	(37.2)	44	(17.9)	
Accompaniment to school (n = 647)							
IM	299	(46.2)	176	(40.2)	123	(58.9)	< 0.001^a^
Accompanied	348	(53.8)	262	(59.8)	86	(41.1)	

*SD* standard deviation

^a^p < 0.001

### Data collection

The children and adolescents completed the same questionnaire in both countries during school hours in approximately 40 min and after provided a written and oral consent to participate. The research team and teachers carefully explained how the questionnaire should be completed and helped to resolve doubts. The parental questionnaire was delivered to children and completed at home by the parents. Additionally, parents previously signed an informed consent form that explained the objectives and characteristics of this study and allowed their offspring to participate in accordance with the Declaration of Helsinki [33].

### Measures

#### Sociodemographic characteristics

The parents and their offspring self-reported their sociodemographic characteristics, including the birth date, age, school grade, gender, birth country, and full postal address [34, 35]. The parental questionnaire included sociodemographic variables, such as salary/month (nine categories each 500 € from 0 € to > 5000 €), dichotomised in < 1000 € and ≥ 1000 € according to the minimum salary in Spain which is around 1000 euros and it has been homogenized for the Chilean sample. Additionally, the parents reported their highest educational level (response options: *no study, primary school, secondary school, bachelor’s degree, professional, university degree*), dichotomised into low-medium education *(no study, primary school, secondary school, bachelor’s degree)* or higher education *(professional or university degree)*.

The socioeconomic level was assessed using the Family Affluence Scale II (FAS II) [36]. The sum of the scores of the 4 questions concerning FAS II was calculated. The participants were classified into three categories regarding FAS: *low level* [0 to 3 points], *medium level* [4 to 5 points] and *high level* [6 to 7 points] [37]. Additionally, car availability in the family was used as an independent variable that according to the evidence affects the ACS and the IM to school.

The distance from home to work and to school were categorised as follows: < *0.5 km; 0.5 km to* < *1 km; 1 km to* < *2 km; 2 km to* < *3 km; 3 km to* < *5 km and* ≥ *5 km*. Furthermore, the active commuting distance was categorized as < *1 km and* ≥ *1 km* according to previous studies [37, 38].

#### Parental physical activity

The International Physical Activity Questionnaire (IPAQ, short version) was used to evaluate the parents’ PA levels. IPAQ has been validated in 12 countries in adults and shows acceptable psychometric properties to measure the PA levels in one week [39] In Spanish population IPAQ has showed acceptable validity for total and vigorous PA and good reliability coefficients for application [40]. Furthermore, IPAQ determines different intensity categories according to METs (Metabolic Equivalent Tasks), such as sedentary (< 1.5 METs), light PA (1.5–3 METs), moderate PA (3–6 METs), and vigorous PA (> 6 METs) in minutes per week. Regarding the international recommendations for adults (≥ 150 min of MVPA per week), the parents were classified as meeting MVPA recommendations (i.e., physically active) and not meeting MVPA recommendations (i.e., physically inactive) [2].

#### Active commuting

The questions about the parents’ mode of commuting to work have undergone a thorough review process [41]. The questions about ACS have been validated [34] and its reliability and feasibility have been verified in the Spanish population [42] and Chilean youth [43]. The usual mode of commuting was categorized as “active” when the parents/children went walking or bike and “passive” when the parents/children went using a motorised mode (car, motorcycle, public bus, metro/train). Finally, “Other mode” was excluded from the analysis because the responses could not be classified (two cases in parents and 11 in offspring’s). The final variable to analyse was the usual active mode of commuting to school.

#### Independent mobility to school

The accompaniment mode to and from school was self-reported by participants following a previous validated and reliable School Travel Survey [44] in children. The questions were as follows: *“Who do you go to the school with? Who do you come back from the school with?”.* The possible answers were as follows: *alone, siblings, friends, father, mother, grandparents, neighbours,* and *other*. Based on these questions, the students were categorized as “children with independent mobility” when they went to or from school *alone*, with *siblings*, or *friends* and they were categorized as “accompanied” when they commuted to or from school with their *father, mother, grandparents or neighbours.* Therefore, the final variable of IM included one or two trips to school (to, from or both).

### Data analysis

Descriptive statistics were presented using means and standard deviation for continuous variables and frequency and percentages for categorical variables. To analyse the differences in sociodemographic factors, PA, and mode of commuting to work among parents (i.e., mothers and fathers, and in sociodemographic factors, IM to school, and mode of commuting to school among offspring (children and adolescents), T-test and chi-squared test were used for continuous and categorical variables, respectively.

The gender of the parent who answered the questionnaire was recorded to establish comparisons. To study the association between the parents’ (mother and father separately) sociodemographic, PA, and mode of commuting to work and IM to school, several binary logistic regression models were performed to obtain the odds ratio (OR) and confidence interval (95% CI). It has been decided to analyse separately by gender of parents, following a principle of "gender perspective". IM to school was established as the dependent variable, and each parent’s variable was included as an independent variable in separate models. The results were analyzed separately by country, but not all were calculated due to lack of data. Nor did it show any differences when joining both countries (see Additional file 1). Therefore, the predictors were calculated from a single group (Chile-Spain together). Finally, a stepwise analysis with the variables of interest that yielded previous statistical significance was performed. In the first model, the sociodemographic factors were included. In the second model, the variables of PA and mode of commuting were included. In the third model, a whole model combined with all variables were included. The OR, 95% CI and R^2^ Nagelkerke [45] were obtained in this stepwise. All the analyses were performed using SPSS® v21 (IBM, New York, NY, USA). A value of p < 0.05 indicated statistical significance.

## Results

The parent’s sociodemographic factors, PA, and mode of commuting to work are shown in Table 1. Mothers presented a higher educational level and a lower salary/month than fathers (p < 0.001 and p = 0.004, respectively). Mothers reported having more car availability than fathers (p < 0.001).

Regarding the distance to work, the mothers’ journey was shorter than that of the fathers. More mothers reached the MVPA recommendations (p < 0.001), but no significant differences were found in the mode of commuting to work (p = 0.254) according to parent gender.

The children’s and adolescents’ sociodemographic factors, mode of commuting and mobility to school are shown in Table 2. A significant difference was found, with adolescents showing more IM to school than children (59% vs. 40%, respectively; p < 0.001).

**Fig. 2:**
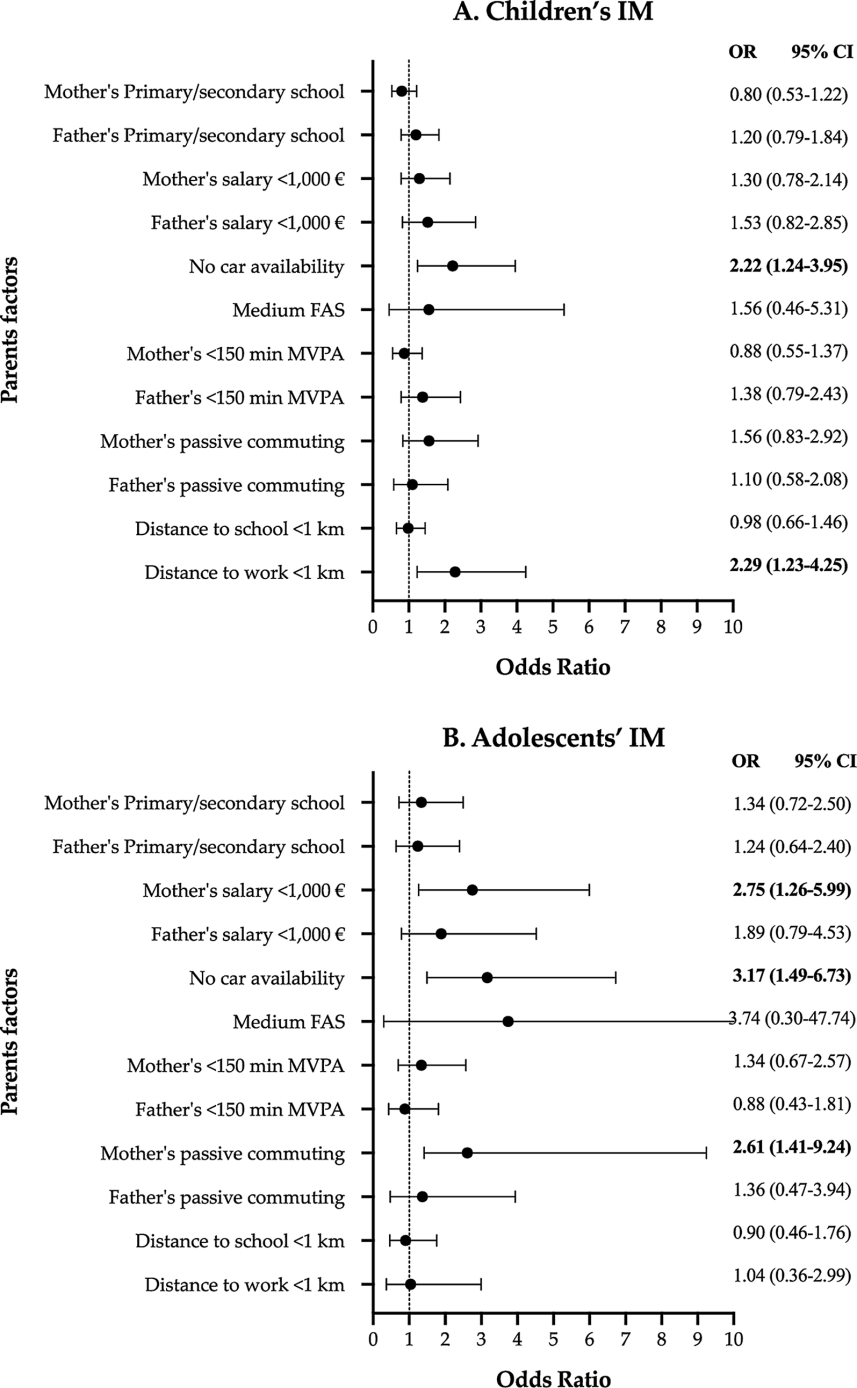
Association between the parents’ sociodemographic, PA, and mode of commuting to work factors and independent mobility in children (**A**) and adolescents (**B**). The reference variable in each model is the opposite category shown in Table 1

Figure 2 shows the associations between sociodemographic factors, PA and mode of commuting to work of parents and independent mobility in children (plot A) and adolescents (plot B). When the parents reported no car availability, the odds for children to independently commute were higher (OR = 2.26; 95% CI = 1.24–3.96). Moreover, a positive association was found between the shorter distance to work and IM (OR = 2.29; 95% CI = 1.23–4.25). In adolescents, three positive associations with IM were presented: mothers' lower salary/month (OR = 2.75; 95% CI = 1.26–5.99), no car availability (OR = 3.17; 95% CI = 1.49–6.73) and mother passive commuting (OR = 2.61; 95% CI = 1.41–9.24).

Finally, stepwise analysis of the sociodemographic, PA, and mode of commuting to work factors of parents as predictors of IM to school in adolescents was performed. Three models were developed using different factors. The combination of the variables produced a single step for each model.

In children (Table 3), the model 1 which included the parents’ sociodemographic factors, the strongest predictor for IM to school was no car availability (OR = 11.26; 95% CI = 1.32–95.85). Model 2 included PA and mode of commuting to work factors, presented weaker predictors for IM to school in children, where less distance to work was positively associated with IM to school (OR = 2.07; 95% CI = 1.14–3.74). Model 3 included sociodemographic, PA, and mode of commuting to work factors. Distance to work was repeatedly identified as a predictor in children (OR = 2.17; 95% CI = 1.10–4.28).

**Table 3:** Sociodemographic, PA and mode of commuting factors of parents as predictors of IM in children

Models	Predictors	*B*	OR	95% CI	*p-*value	R^2^
Model 1	No car availability	2.422	11.26	(1.32–95.85)	0.027	0.058
Model 2	Distance to work < 1 km	0.726	2.07	(1.14–3.74)	0.016	0.024
Model 3	Distance to work < 1 km	0.776	2.17	(1.10–4.28)	0.025	0.026

B: B value; OR: odds ratio; CI: confidence interval; R^2^: Nagelkerke correlation

Model 1: Sociodemographic factors only (i.e., age, educational level, salary/month, car availability, and family affluence scale II)

Model 2: Physical activity and mode of commuting to work factors only (Active or passive commuting to work, distance of commuting to work, and complied to 150-min MVPA)

Model 3: Combination of all the factors, Sociodemographic, physical activity and mode of commuting

In adolescents (Table 4), the model 1 showed that less mother’s salary was the main IM predictor (OR = 6.18; 95% CI = 1.77–21.55). In model 2 was mother’s passive commuting to work (OR = 2.47; 95% CI = 1.02–5.99) and in model 3 was no car availability (OR = 6.53; 95% CI = 2.23–19.08), which was the second strongest predictor from all the models.

**Table 4:** Sociodemographic, PA and mode of commuting factors of parents as predictors of IM in adolescents

Models	Predictors	*B*	OR	95% CI	*p-*value	R^2^
Model 1	Mother salary/month < 1.000 €	1.821	6.18	(1.77–21.55)	0.004	0.197
Model 2	Mother passive commuting to work	0.726	2.47	(1.02–5.99)	0.045	0.038
Model 3	No car availability	1.876	6.53	(2.23–19.08)	0.001	0.173

*B* B value, *OR* odds ratio, *CI* confidence interval, *R*^*2*^ Nagelkerke correlation

Model 1: Sociodemographic factors only (i.e., age, educational level, salary/month, car availability, and family affluence scale II)

Model 2: Physical activity and mode of commuting to work factors only (Active or passive commuting to work, distance of commuting to work, and complied to 150-min MVPA)

Model 3: Combination of all the factors, Sociodemographic, physical activity and mode of commuting

## Discussion

This study mainly aimed to analyse whether parental sociodemographic factors, parents’ PA, and active commuting to work serve as predictors of IM to school in children and adolescents. We found that independent mobility was higher in adolescents than in children. Additionally, a shorter distance from home to work of the parents and no car availability were the main predictors of IM.

### Independent mobility to school

According to our results, the prevalence of IM to school was 40.2% in children and 58.9% in adolescents. This can be explained by the lesser independence that parents give to children [19]. It is important to increase IM, particularly in children, because moving autonomously contribute to children to develop their physical, mental, cognitive performance and to build social relationships [46]. Additionally, the parents’ role is fundamental for developing children’s autonomy and IM [47]. Furthermore, parents can act in a controlling manner, leading to their children’s conduct being judged according to adult criteria [8, 48]. According to other studies about different destinations, 25% IM was found in England [24], 25.4% in New Zealand [49], and 31% in Australia [50]. The cause may be related to the long distances that must be travelled in these countries to commute to schools and the decrease observed in developed countries in the last 20 years [24]. Nevertheless, other European countries showed higher values of IM (Germany: 76%; Finland: 65%) than those in the current study. Additionally, countries with school policies to ensure the safety of transportation to school have been positively associated with ACS [51]. This finding might be due to social cohesion present in developed countries—the degree of association between adults and children living in the same neighbourhood [52]. Parents with larger social networks and a positive perception of social cohesion tend to grant higher IM licenses [24, 53].

According to the scientific literature, adolescents show higher IM than children [30, 54]. This finding could be explained by the high degree of autonomy and independence of adolescents who no longer depend heavily on parents for commuting [55]. Additionally, the parents of children might have a greater fear of danger and strangers when their offspring is travelling alone compared with their older children.

As future implications, it is necessary to develop local intervention programmes and case studies promoting IM, particularly in countries with low levels of IM and ACS as well as Chile. For this to occur, the educational institutions must identify the determinants of each country and context that inhibit (i.e., longer distance, car use, parental fears and restrictions) or promote IM (i.e., safe environment, shorter distance, parental active commuting), and better understand the problem. Some proposals that can compensate for the non-modifiable sociodemographic variables are, educate parents about the importance of developing independence in children, implement interventions that improve the safety of the routes to school, reduce the speed of traffic in school zones and provide to children of knowledge and practices about road safety that increases their self-confidence and consequently, the IM.

### Association of parents’ sociodemographic status on independent mobility to school

Our results showed a positive association between a lower mother’ salary/month and IM to school in adolescents, but not in children. This could be related to a lower family income influencing the choice of a school closer to home and regularly living in a smaller neighbourhood, facilitating IM to school. In addition, socioeconomic status could be highly related to the neighbourhood quality and safety where children live, therefore parents could give less licenses to move independently. Unlike, high-status households also more often include two employed parents, multiple cars, and the selection of a private and distant school, facilitating dropping off a child at school by car [56]. Several studies have found that children living in high-income households and with home ownership, commute less independently [57–59]. In the case of children, where there was no association, it is important to mention that they commute to school mostly due to a greater distance than adolescents (See Table 2), therefore the association is lost. On the other hand, fathers are less responsible for children's school activities, therefore they are less influential [60].

In the current study, no car availability was associated with IM to school in children and adolescents. Also, it was the main predictor of IM in adolescents, among all the factors of parents studied. Furthermore, adolescents were reported to prefer being driven to school than using another active mode of commuting [61]. These findings are essential because these preferences of young people to use a passive mode of commuting could be transferred to adult life [62]. In another study, where the parents were asked to explain why they drove their children to school by car, they expressed three reasons: distance to destination, compatibility with their own destinations and time savings [63]. Thus, longer commuting distances caused by urban sprawl limit children’s mobility opportunities [64]. In this regard, a study conducted in Spain in school children aged 9–12 years [62] observed a higher parental perception of driving being more convenient than walking their children to school. Parents’ convenience has been previously reported as one of the main barriers to ACS [65, 66] and could be a barrier also to IM to school. Therefore, parents who own cars should consider implementing their use only when it is necessary. Children commuting independently and actively instead of using motorised transport provide further benefits such as low monetary costs for travelling and environmental sustainability through reduced car use, less traffic volume, and air pollution [24]. This study demonstrates the greater influence of parental sociodemographic factors on IM to school than PA or active commuting by parents. This discovery leads us to believe that interventions to increase parenting active behaviours may be less effective in modelling their children's active behaviour. However, interventions to increase parental support with the objective of increasing IM could be more effective in families where there are more sociodemographic factors that hinder IM in children and adolescents.

### Effects of parents’ physical activity and active commuting on independent mobility

A weak positive association was found between parents’ PA factors and active commuting with IM. Only the mother’s passive mode of commuting was positively associated with IM. According to our knowledge, the only study that has investigated the association between parents’ PA and IM [67] found a weak positive association. This finding could be explained by IM depending not only on parent’s active lifestyle but also on other sociodemographic and availability factors of parents that can influence their children.

Regarding the mode of commuting to work, according to our results, the shorter distance from home to work was positively associated with high IM. A previous study showed that mothers with longer working hours or longer commuting distances are less likely to escort their children [28]. Therefore, children must find another way to travel, which is usually commuting alone.

In adolescents, a positive association was found between the mothers’ passive commuting to work and IM. This finding could be explained by the mothers travelling further using passive modes of commuting such as cars. Additionally, mothers accompany their teenage offspring less. Strategies to increase ACS and to other destinations may be important to increase IM in children and adolescents [68]. Thus, it is important to continue implementing schools’ interventions based on socioecological models that include their close connections, parents and their factors to promote ACS and IM to school in children and adolescents. Likewise, it is necessary to involve families and the whole school community to provide tools to facilitate IM motivating them to active commuting and less use of the car.

### Strengths and limitations

The main strengths are the high sample number of parents and their offspring stands out as strengths, reaching 1,368 participants. The data from two Spanish-speaking countries were enrolled with their respective language adaptations. Additionally, the novelty of the study was to have included sociodemographic and PA variables in the same model, providing new evidence on parents and their offspring.

The main limitation of the study was the cross-sectional design and, therefore, no cause-and-effect relationship can be established in the associations found. A longitudinal study would be required to determine the direction of the relationship. A relevant loss of sample data occurred regarding the initial data collection because the questionnaires were incomplete. Moreover, no imputation data process was performed for avoid some interpretation error in the sociodemographic variables. Non-randomized sample was included; therefore, it is not possible to generalize the results to the entire population. A self-reported questionnaire was used that has a lower objectivity to determine PA than devices such as accelerometers. The questionnaire asked about the socioeconomic level separately by gender of the parents, which limits calculating the level of family income.

## Conclusions

Our results confirm higher IM to school in adolescents than in children. Likewise, the sociodemographic factors of parents, such as a low salary/month and no car availability, are positively associated with IM to school in children and adolescents. Additionally, these parental factors are more significant predictors of IM to school than PA and the parental active commuting to work. Moreover, a smaller distance of commuting to work for mothers was associated with IM to school. Finally, parental behaviour was not associated with IM to school, but the family economic context could influence and predict IM in children and adolescents.

## Supplementary Information

**Additional file 1: Table S1.** Predictors separated by country.

## Data Availability

Not applicable.

## References

[1] Poitras VJ, Gray CE, Borghese MM, Carson V, Chaput JP, Janssen I (2016). Systematic review of the relationships between objectively measured physical activity and health indicators in school-aged children and youth. Appl Physiol Nutr Metab.

[2] Bull FC, Al-Ansari SS, Biddle S, Borodulin K, Buman MP, Cardon G (2020). World Health Organization 2020 guidelines on physical activity and sedentary behaviour. Br J Sports Med.

[3] Piercy KL, Troiano RP, Ballard RM (2018). The physical activity guidelines for Americans. JAMA.

[4] Aubert S, Barnes JD, Abdeta C, Abi Nader P, Adeniyi AF, Aguilar-Farias N (2018). Global matrix 3.0 physical activity report card grades for children and youth: results and analysis from 49 countries. J Phys Act Health..

[5] Guthold R, Stevens GA, Riley LM, Bull FC (2020). Global trends in insufficient physical activity among adolescents: a pooled analysis of 298 population-based surveys with 1· 6 million participants. Lancet Child Adolesc Health.

[6] Vancampfort D, Van Damme T, Firth J, Smith L, Stubbs B, Rosenbaum S (2019). Correlates of physical activity among 142,118 adolescents aged 12–15 years from 48 low-and middle-income countries. Prev Med.

[7] Telama R, Yang X, Leskinen E, kankaanpää A, Hirvensalo M, Tammelin T, et al. Tracking of physical activity from early childhood through youth into adulthood. Med Sci Sports Exerc. 2014;46(5):955–62. 10.1249/MSS.0000000000000181. 10.1249/MSS.0000000000000181 24121247

[8] Sallis JF, Cervero RB, Ascher W, Henderson KA, Kraft MK, Kerr J (2006). An ecological approach to creating active living communities. Annu Rev Public Health..

[9] Chillón P, Ortega FB, Ruiz JR, Veidebaum T, Oja L, Mäestu J (2010). Active commuting to school in children and adolescents: an opportunity to increase physical activity and fitness. Scand J Public Health.

[10] McMillan T, Day K, Boarnet M, Alfonzo M, Anderson C (2006). Johnny walks to school— does Jane? Sex differences in children’s active travel to school. Child Youth Environ.

[11] Tranter P, Whitelegg J (1994). Children’s travel behaviours in Canberra: Car dependent lifestyles in a low density city. J Transp Geogr.

[12] Schoeppe S, Duncan MJ, Badland H, Oliver M, Curtis C (2013). Associations of children’s independent mobility and active travel with physical activity, sedentary behaviour and weight status: a systematic review. J Sci Med Sport.

[13] Stone MR, Faulkner GEJ, Mitra R, Buliung RN (2014). The freedom to explore: examining the influence of independent mobility on weekday, weekend and after-school physical activity behaviour in children living in urban and inner-suburban neighbourhoods of varying socioeconomic status. Int J Behav Nutr Phys Act.

[14] Marques EA, Pizarro AI, Mota J, Santos PM (2014). Independent mobility and its relationship with moderate to vigorous physical activity in middle school portuguese boys and girls. J Phys Act Health.

[15] Cohen R (2013). The development of spatial cognition.

[16] Freeman C, Quigg R (2009). Children's lives: a Dunedin study. Childrenz Issues.

[17] Oliver M, Witten K, Kearns RA, Mavoa S, Badland HM, Carroll P (2011). Kids in the city study: research design and methodology. BMC Public Health.

[18] Herrador-Colmenero M, Villa-González E, Chillón P (2017). Children who commute to school unaccompanied have greater autonomy and perceptions of safety. Acta Paediatr.

[19] Riazi NA, Faulkner G (2018). Children’s independent mobility. Children's Active Transport.

[20] Janssen I, Ferrao T, King N (2016). Individual, family, and neighborhood correlates of independent mobility among 7 to 11-year-olds. Prev Med Rep.

[21] Ghekiere A, Deforche B, Carver A, Mertens L, de Geus B, Clarys P (2017). Insights into children’s independent mobility for transportation cycling—which socio-ecological factors matter?. J Sci Med Sport.

[22] Cordovil R, Lopes F, Neto C (2015). Children’s (in) dependent mobility in Portugal. J Sci Med Sport.

[23] Foster S, Villanueva K, Wood L, Christian H, Giles-Corti B (2014). The impact of parents’ fear of strangers and perceptions of informal social control on children's independent mobility. Health Place.

[24] Marzi I, Reimers AK (2018). Children’s independent mobility: Current knowledge, future directions, and public health implications. Int J Environ Res Public Health.

[25] Veitch J, Salmon J, Ball K (2017). Children’s active free play in local neighborhoods: a behavioral mapping study. Health Educ Res.

[26] Lin EY, Witten K, Smith M, Carroll P, Asiasiga L, Badland H (2017). Social and built-environment factors related to children’s independent mobility: the importance of neighbourhood cohesion and connectedness. Health Place.

[27] Kyttä M, Hirvonen J, Rudner J, Pirjola I, Laatikainen T. The last free-range children? Children’s independent mobility in Finland in the 1990s and 2010s. J Transport Geogr. 2015;47:1–2. 10.1016/j.jtrangeo.2015.07.004.

[28] Shaw B, Bicket M, Elliott B, Fagan-Watson B, Mocca E, Hillman M. Children’s independent mobility: an international comparison and recommendations for action. 2015. https://www.nuffieldfoundation.org/sites/default/files/files/7350_PSI_Report_CIM_final.pdf.

[29] Fyhri A, Hjorthol R, Mackett RL, Fotel TN, Kytta M (2011). Children’s active travel and independent mobility in four countries: development, social contributing trends and measures. Transp Policy..

[30] He SY, Giuliano G (2017). Factors affecting children’s journeys to school: a joint escort-mode choice model. Transportation.

[31] Huertas-Delgado FJ, Mertens L, Chillon P, Van Dyck D (2018). Parents’ and adolescents’ perception of traffic-and crime-related safety as correlates of independent mobility among Belgian adolescents. PLoS ONE.

[32] Bringolf-Isler B, Schindler C, Kayser B, Suggs LS, Probst-Hensch N, SOPHYA Study Group. Objectively measured physical activity in population-representative parent-child pairs: Parental modelling matters and is context-specific. BMC Public Health. 2018;18(1):1024. 10.1186/s12889-018-5949-9. 10.1186/s12889-018-5949-9 PMC6098593 30119661

[33] World Medical Association. World medical association declaration of Helsinki; 2010. https://www.wma.net/what-we-do/medical-ethics/declaration-of-helsinki/doh-oct2008/.

[34] Chillón P, Herrador-Colmenero M, Migueles JH, Cabanas-Sánchez V, Fernández-Santos JR, Veiga ÓL (2017). Convergent validation of a questionnaire to assess the mode and frequency of commuting to and from school. Scand J Public Health.

[35] Aranda-Balboa MJ, Fernández M, Villa-González E, Murillo-Pardo B, Segura-Díaz JM, Saucedo-Araujo RG (2020). Psychometric characteristics of a commuting-to-school behaviour questionnaire for families. Int J Environ Res Public Health.

[36] Torsheim T, Cavallo F, Levin KA, Schnohr C, Mazur J, Niclasen B (2016). Psychometric validation of the revised family affluence scale: a latent variable approach. Child Indic Res..

[37] Rodriguez-Rodriguez F, Cristi-Montero C, Celis-Morales C, Escobar-Gómez D, Chillón P (2017). Impact of distance on mode of active commuting in Chilean children and adolescents. Int J Environ Res Public Health.

[38] Campos-Sánchez FS, Abarca-Álvarez FJ, Molina-García J, Chillón P (2020). A Gis-based method for analysing the association between school-built environment and home-school route measures with active commuting to school in urban children and adolescents. Int J Environ Res Public Health.

[39] Craig CL, Marshall AL, Sjöström M, Bauman AE, Boothe ML, Ainsworth B (2003). International physical activity questionnaire: 12-country reliability and validity. Med Sci Sports Exerc.

[40] Roman-Viñas B, Serra-Majem L, Hagströmer M, Ribas-Barba L, Sjöström M, Segura-Cardona R (2010). International physical activity questionnaire: reliability and validity in a Spanish population. Eur J Sport Sci.

[41] Herrador-Colmenero M, Ruiz JR, Ortega FB, Segura-Jiménez V, Álvarez-Gallardo IC, Camiletti-Moirón D (2015). Reliability of the ALPHA environmental questionnaire and its association with physical activity in female fibromyalgia patients: the al-Ándalus project. J Sports Sci.

[42] Segura-Díaz JM, Rojas-Jiménez Á, Barranco-Ruiz Y, Murillo-Pardo B, Saucedo-Araujo RG (2020). Feasibility and reliability of a questionnaire to assess the mode, frequency, distance and time of commuting to and from school: the PACO study. Int J Environ Res Public Health..

[43] Escobar-Gómez D, Rodríguez-Rodríguez F, Villa-González E, Esteban-Cornejo I, Chillón P (2020). Fiabilidad y viabilidad de un cuestionario autorreportado sobre el modo tiempo y distancia de desplazamiento en niños y adolescents. Retos.

[44] Evenson KR, Catellier DJ, Gill K, Ondrak KS, McMurray RG (2008). Calibration of two objective measures of physical activity for children. J Sports Sci..

[45] Nagelkerke NJ (1991). A note on a general definition of the coefficient of determination. Biometrika.

[46] Ayllón E, Moyano N, Aibar-Solana A, Salamanca A, Bañares L (2020). Independent mobility to school and Spanish children: go, return, or both?. Chil Geogr..

[47] Ayllón E, Moyano N, Lozano A, Cava MJ (2019). Parents’ willingness and perception of children’s autonomy as predictors of greater independent mobility to school. Int J Environ Res Public Health.

[48] Villanueva K, Giles-Corti B, Bulsara M, Trapp G, Timperio A, McCormack G (2014). Does the walkability of neighbourhoods affect children's independent mobility, independent of parental, socio-cultural and individual factors?. Chil Geogr.

[49] Jelleyman C, McPhee J, Brussoni M, Bundy A, Duncan S (2019). A cross-sectional description of parental perceptions and practices related to risky play and independent mobility in children: The New Zealand state of play survey. Int J Environ Res Public Health.

[50] Schoeppe S, Tranter P, Duncan MJ, Curtis C, Carver A, Malone K (2016). Australian children's independent mobility levels: secondary analyses of cross-sectional data between 1991 and 2012. Chil Geogr.

[51] Hollein T, Vašíčková J, Bucksch J, Kalman M, Sigmundová D, van Dijk JP (2017). School physical activity policies and active transport to school among pupils in the Czech Republic. J Transp Health.

[52] Tyagi M, Raheja G (2020). Indian parents’ perception of children's independent mobility in urban neighbourhoods: a case study of Delhi. Chil Geogr..

[53] Porskamp T, Ergler C, Pilot E, Sushama P, Mandic S (2019). The Importance of social capital for young people’s active transport and independent mobility in rural Otago New Zealand. Health Place.

[54] Vlaar J, Brussoni M, Janssen I, Mâsse LC (2019). Roaming the neighbourhood: influences of independent mobility parenting practices and parental perceived environment on children’s territorial range. Int J Environ Res Public Health.

[55] Garcia-Cervantes L, Rodriguez-Romo G, Esteban-Cornejo I, Cabanas-Sanchez V, Delgado-Alfonso A, Castro-Pinero J (2016). Perceived environment in relation to objective and self-reported physical activity in Spanish youth. The UP&DOWN study. J Sports Sci.

[56] Scheiner J, Huber O, Lohmüller S (2019). Children's independent travel to and from primary school: evidence from a suburban town in Germany. Transport Res Part A.

[57] Hsu H-P, Saphores J-D (2014). Impacts of parental gender and attitudes on children's school travel mode and parental chauffeuring behavior: results for California based on the 2009 National Household Travel Survey. Transportation.

[58] Ermagun A, Samimi A (2016). How are children accompanied to school?. J Urban Plan Dev.

[59] Scheiner J (2016). School trips in Germany: gendered escorting practices. Transport Res Part A.

[60] Rodríguez-Rodríguez F, Huertas-Delgado FJ, Barranco-Ruiz Y, Aranda-Balboa MJ, Chillón P (2020). Are the parents’ and their children’s physical activity and mode of commuting associated? Analysis by gender and age group. Int J Environ Res Public Health.

[61] Mandic S, Hopkins D, García Bengoechea E, Flaherly Ch, Williams J (2017). Adolescents' perceptions of cycling versus walking to school: Understanding the New Zealand context. J Transp Health.

[62] Solana AA, Mandic S, Lanaspa EG, Gallardo LO, Casterad JZ (2018). Parental barriers to active commuting to school in children: does parental gender matter?. J Transp Health.

[63] McDonald NC, Aalborg AE (2009). Why parents drive children to school: implications for safe routes to school programs. J Am Plan Assoc.

[64] Marzi I, Demetriou Y, Reimers AK (2018). Social and physical environmental correlates of independent mobility in children: a systematic review taking sex/gender differences into account. Int J Health Geogr.

[65] Faulkner GEJ, Richichi V, Buliung RN, Fusco C, Moola F (2010). What’s “quickest and easiest?": parental decision making about school trip mode. Int J Behav Nutr Phys Act.

[66] Trapp GSA, Giles-Corti B, Christian HE, Bulsara M, Timperio AF, McCormack GR (2011). On your bike! a cross-sectional study of the individual, social and environmental correlates of cycling to school. Int J Behav Nutr Phys Act.

[67] Santos MP, Pizarro AN, Mota J, Marques EA (2013). Parental physical activity, safety perceptions and children’s independent mobility. BMC Public Health.

[68] Huertas-Delgado FJ, Herrador-Colmenero M, Villa-González E, Aranda-Balboa MJ, Cáceres MV, Mandic S (2017). Parental perceptions of barriers to active commuting to school in Spanish children and adolescents. Eur J Public Health.

