# Supplementary material for: Parent’s sociodemographic factors, physical activity and active commuting are predictors of independent mobility to school
Source: Int J Health Geogr. 2021 Jun 6;20:26. doi: 10.1186/s12942-021-00280-2 (PMC8180041; doi:10.1186/s12942-021-00280-2)
Supplement: Supplementary file 1 — Additional file 1: Table S1. Predictors separated by country. [file 12942_2021_280_MOESM1_ESM.docx]

Additional file 1

**Table 4**. Predictors separated by country

| **Models** | **Predictors** | ***B*** | **OR** | **95% CI** | ***p-*value** | **R^2^** |
| --- | --- | --- | --- | --- | --- | --- |
| **Model 1** |  |  |  |  |  |  |
| Chilean Children | No car availability | 2.996 | 20.00 | (1.42–282.45) | 0.027 | 0.449 |
|  |  |  |  |  |  |  |
| Chilean Adolescents | ***not calculated due to lack of data*** |  |  |  |  |  |
|  |  |  |  |  |  |  |
| Spanish Children | No car availability | 2.440 | 11.47 | (1.35-97.63) | 0.026 | 0.059 |
|  |  |  |  |  |  |  |
| Spanish Adolescents | ***not calculated due to lack of data*** |  |  |  |  |  |
|  |  |  |  |  |  |  |
| **Model 2** |  |  |  |  |  |  |
| Chilean Children | ***not calculated due to lack of data*** |  |  |  |  |  |
|  |  |  |  |  |  |  |
| Chilean Adolescents | ***not calculated due to lack of data*** |  |  |  |  |  |
|  |  |  |  |  |  |  |
| Spanish Children | Distance to work < 1 km | 0.868 | 2.38 | (1.19–477) | 0.014 | 0.036 |
|  |  |  |  |  |  |  |
| Spanish Adolescents | ***not calculated due to lack of data*** |  |  |  |  |  |
|  |  |  |  |  |  |  |
| **Model 3** |  |  |  |  |  |  |
| Chilean Children | ***not calculated due to lack of data*** |  |  |  |  |  |
|  |  |  |  |  |  |  |
| Chilean Adolescents | ***not calculated due to lack of data*** |  |  |  |  |  |
|  |  |  |  |  |  |  |
| Spanish Children | No car availability | 2.154 | 8.622 | (0.937-79.359) | 0.057 | 0.044 |
|  |  |  |  |  |  |  |
| Spanish Adolescents | ***not calculated due to lack of data*** |  |  |  |  |  |
